# Supplementary material for: Proteo-Molecular Investigation of Cultivated Rice, Wild Rice, and Barley Provides Clues of Defense Responses against Rhizoctonia solani Infection
Source: Bioengineering (Basel). 2022 Oct 20;9(10):589. doi: 10.3390/bioengineering9100589 (PMC9598808; doi:10.3390/bioengineering9100589)
Supplement: Supplementary file 1 [file bioengineering-09-00589-s001.zip › bioengineering-1914103-supplementary by author/bioengineering-1914103 Supplementary.pdf]

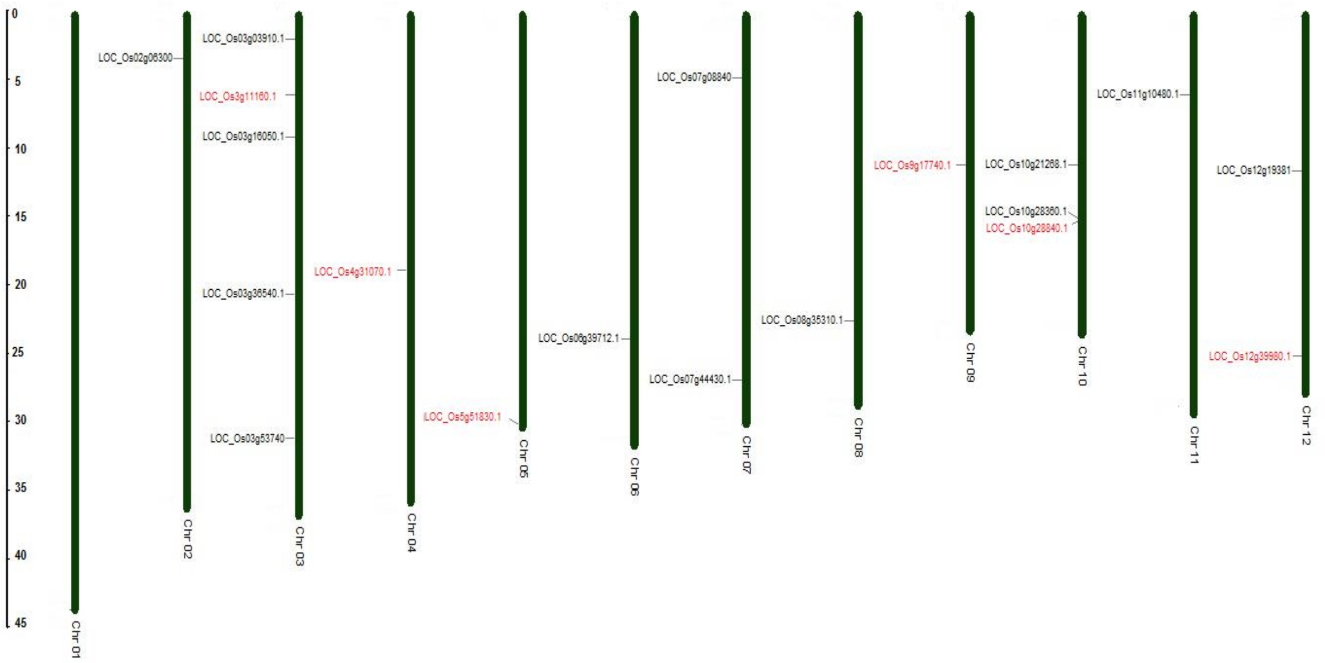

Figure S1. Chromosomal location of genes encoding differentially expressed proteins of cultivated rice cv. Pusa Basmati-1 (black colour) and wild rice accession *O. grandiglumis* (red colour). The Proteins were mapped to the rice genome according to the locus of the markers.

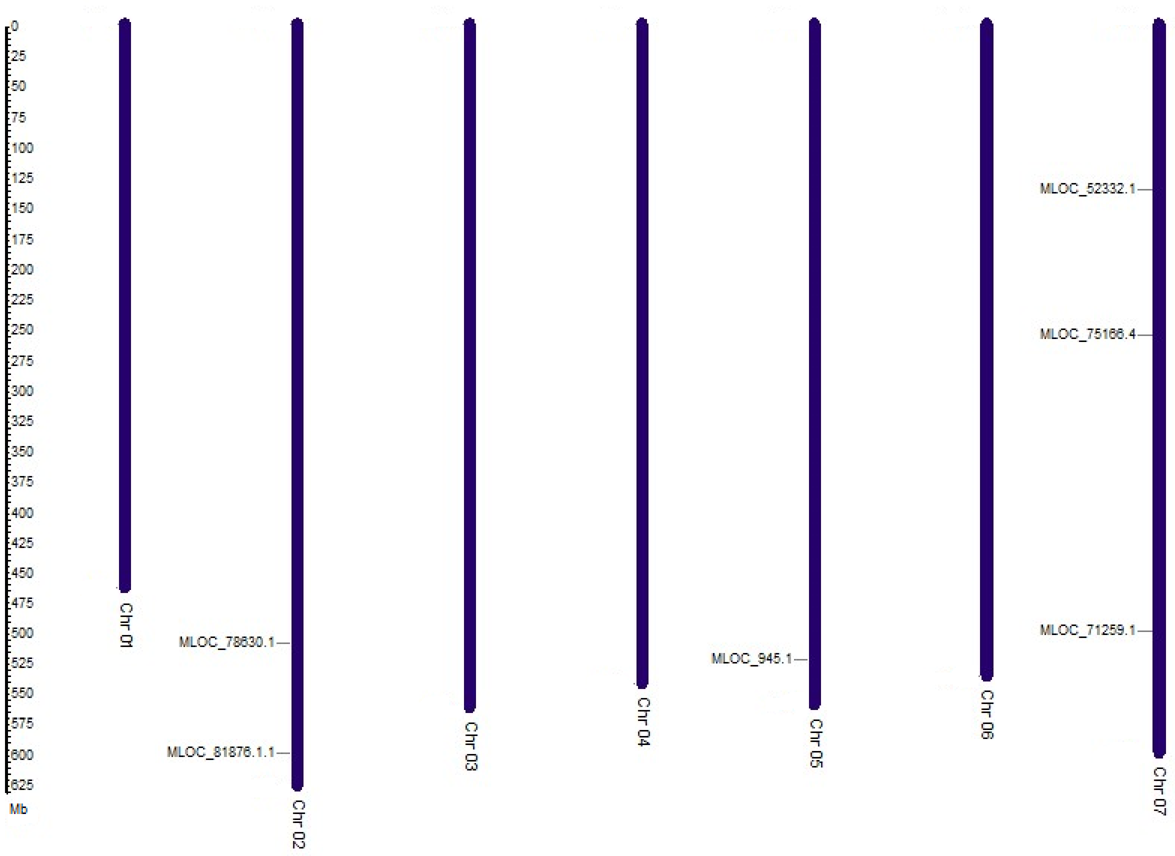

Figure S2. Chromosomal location of genes encoding differentially expressed proteins of barley cv. NDB-1445 . The proteins mapped to the barley genome according to the locus of the markers.
